# Never Settling Down: Frequent Changes in Sex Chromosomes

**DOI:** 10.1371/journal.pbio.1002077

**Published:** 2015-04-16

**Authors:** Kevin H-C Wei, Daniel A. Barbash

**Affiliations:** Department of Molecular Biology and Genetics, Cornell University, Ithaca, New York

## Abstract

A new study reveals multiple dramatic changes in sex chromosome structure and identity in flies; such transitions are accompanied by a series of genomic events that affect chromosome biology, gene regulation, and sex determination. See the accompanying Research Article.

## Overview

Almost universally, animals have two sexes: male and female. In most cases, the genetic basis of sexual differences can be traced to the sex chromosomes. In humans, fruit flies, and many other animals, females have two X chromosomes and males have one X and one Y. This karyotype has independently emerged multiple times in different animal lineages, but the sex chromosomes typically follow a similar trajectory whereby the two sex chromosomes differentiate into a specialized X and a degenerate Y. However, over short evolutionary times, sex chromosomes were thought to be relatively stable, with only infrequent changes such as fusion of sex chromosomes and autosomes. But recent work, including that reported in this issue by Vicoso and Bachtrog, is challenging this notion. The authors use DNA sequencing and mapping across many Diptera (fly) species to identify their sex chromosomes and discover numerous major changes in sex chromosome structure and identity among 37 species. These studies raise important questions of how gene dosage and regulation are maintained as karyotypes change, and what evolutionary forces drive the continual changes in sex chromosome identity.

## Sex Determination Is Labile

Most animals come in male and female forms with many obvious visible and physiological differences. Cytologists long ago noticed that male and female karyotypes also often differ, with one sex having two distinct or heteromorphic sex chromosomes and the other having two copies of a single monomorphic sex chromosome. In many organisms including mammals and Drosophila, males are heterogametic with the X and Y chromosomes, while females are homogametic with two X chromosomes. In birds, butterflies, and other species, the opposite pattern is found where females are heterogametic (ZW chromosomes) and males are homogametic (ZZ chromosomes). It was also appreciated long ago that the genetic basis of sex determination is remarkably variable. For example, although mammals and Drosophila have the same sex chromosome karyotypes, sex determination depends on a dominant Y-linked male determiner in the former and a system of dosage-dependent, X-linked female determiners in the latter [1,2]. Then there are curious cases like houseflies, where different populations have been found having male determiners on different chromosomes [3]. Beyond even this variation are some amphibians and reptiles that determine sex based on environmental signals [4].

These observations raise some fundamental questions that often arise in many other contexts when thinking about evolution. How do regulatory pathways controlling conserved phenotypes evolve? How do evolutionary transitions occur? The dissection of sex determination regulatory pathways has demonstrated that they display a mix of highly conserved and evolutionarily labile components. For example, the primary regulator of sex determination and dosage compensation in Drosophila, the *Sex-lethal* gene, does not show sex-specific regulation in other flies or insects and is thus unlikely to play a role in sex determination outside the Drosophilidae family [5]. Two of its downstream regulatory targets, the *transformer* and *doublesex* genes, however, appear to determine sex throughout insects [6]. Looking more broadly, *doublesex*-related genes regulate sexual differentiation in a very wide range of invertebrates and vertebrates [7]. A picture thus emerges of a deeply conserved terminal regulator upon which upstream control genes have been added on during evolution.

**Fig 1:**
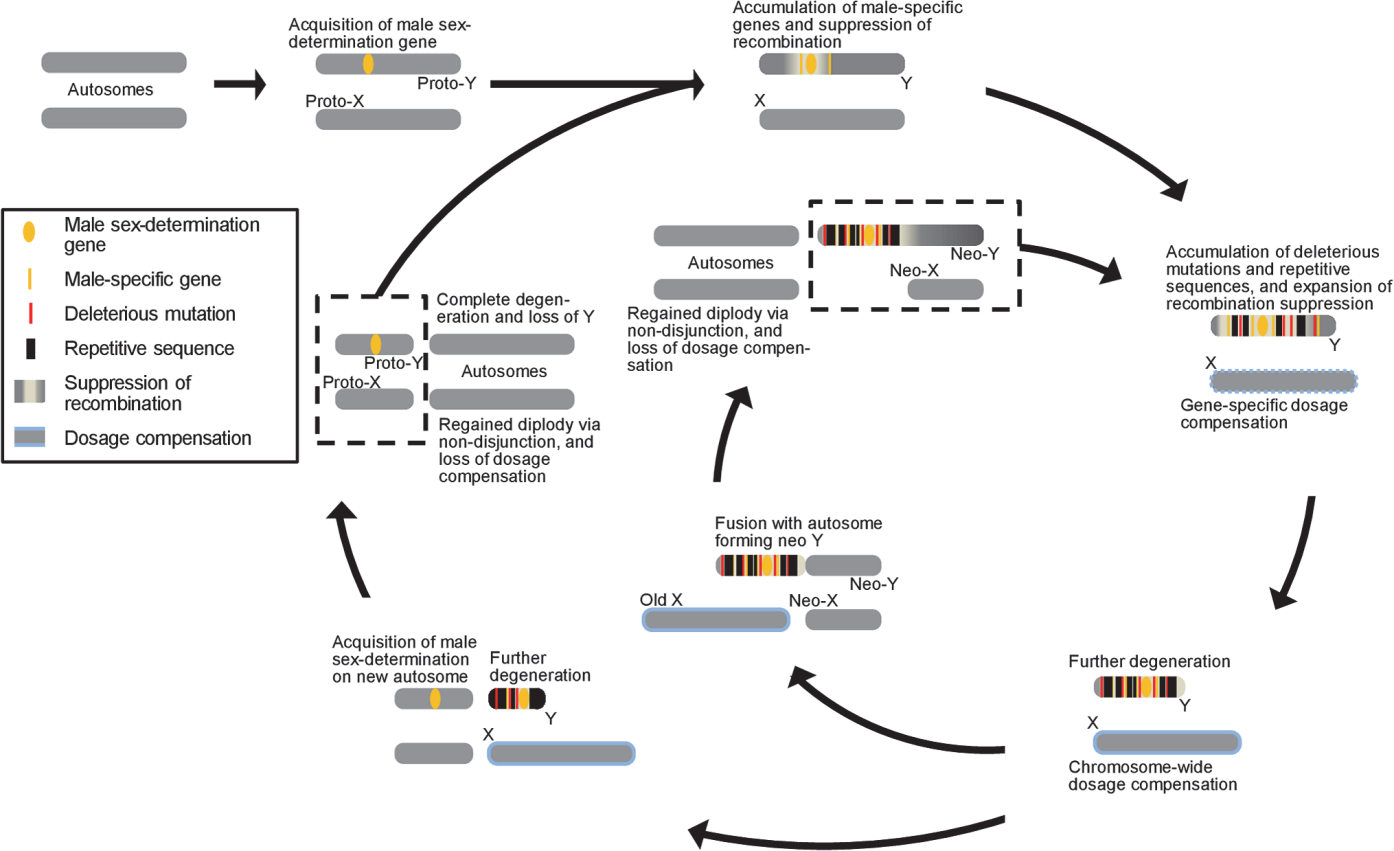
The dynamic cycle and multiple pathways of sex-chromosome evolution. Heteromorphic sex chromosomes evolve from a pair of autosomes and typically follow a trajectory of X specialization and Y degeneration. New sex chromosomes can evolve by either chromosomal fusions or acquisition of new sex-determination genes. Y and X events are indicated above and below the chromosomes, respectively.

What about the evolution of the sex chromosomes? Heteromorphic sex chromosomes are thought to evolve from a pair of homologous autosomes (Fig. 1). Differentiation first begins when one of the two homologs acquires a sex-determining gene either through a de novo mutation or transposition from a different chromosome. If a male-determining gene is inserted, the former autosome now becomes a proto–Y chromosome and will reside exclusively in males. Alleles closely linked to the male-determining gene will also be largely restricted to males because they will rarely be separated by recombination. This sex-specific restriction opens the potential for closely linked alleles to evolve phenotypes that are beneficial only to males; some such alleles may even have pleiotropic deleterious phenotypes if expressed in females. Because crossing over would now create recombinant individuals carrying deleterious combinations of genes favoring opposing sexes, selection will favor the further reduction of recombination around the male-determining gene. Suppression of recombination then permits additional accumulation of male-beneficial genes on the proto-Y, and as the formerly homologous chromosomes become more differentiated, recombination becomes suppressed in larger areas.

However, the cessation of recombination also dooms the heterogametic (Y) chromosome. Recombination is an essential process that separates advantageous and deleterious mutations on the same chromosome, allowing fixation of the former and purging of the latter. In its absence, deleterious mutations irreversibly accumulate, producing a degenerate chromosome containing nonfunctional pseudogenes and replete with noncoding junk DNA. The degeneration is expected to ultimately lead to the complete loss of the Y chromosome.

The homologous X also undergoes specialized evolution. As the Y degenerates and loses gene function, the X becomes effectively haploid in males, while the rest of the genome is diploid. Therefore, the ratio between products of X-linked genes and autosomal genes is halved compared to that of XX females, creating a detrimental imbalance for dosage-sensitive genes.

Multiple dosage compensation strategies have evolved to solve this problem [8]. In Drosophila, the expression of X-linked genes is up-regulated in males via recruitment of a special complex of RNAs and proteins that modifies the male X chromatin state. In contrast, placental mammals solve this imbalance by randomly inactivating one of the X’s in females, via the formation of a heterochromatic silent state known as a Barr body.

These findings have led to the notion that differentiation of sex chromosomes is an evolutionary dead end, as the Y terminally degenerates and the X evolves a specialized chromatin state to achieve dosage compensation. Consistent with this view, all therian mammals share the same X chromosome, thought to have emerged over 180 million years ago. Rarely, a new Y might evolve by transposition or de novo evolution of a new dominant male-determining gene on a former autosome, leading to a repeat of the cycle of sex chromosome differentiation. At other times, differentiated sex chromosomes might fuse with autosomes to create neo-sex chromosomes. However, this process only delays the same fate as the neo-Y will degenerate and the neo-X will become more specialized (Fig. 1).

## Sex Chromosomes Change Often

The karyotypes of most diptera are largely similar, with five large rods and one small dot, named Muller elements A through E and F, respectively. The conserved karyotype seemingly suggests that sex chromosomes are also largely static in these insects, but Vicoso and Bachtrog have shown that this expectation is surprisingly wrong [9]. They devised a simple but effective assay to identify the sex chromosomes from whole genome sequences. In a species with highly differentiated (i.e., old) sex chromosomes, few sequences will remain the same between the X and Y chromosomes because they diverged from a common autosome long ago. Therefore, since males only have one X, the number of DNA sequence reads mapping to it should be half of those mapping to the autosomes. In contrast, the entire genome of females is diploid, so there should be equal mapping coverage of the autosomes and the X. For species with less diverged (i.e., younger) sex chromosomes, the relative number of reads mapping to the X versus autosomes in DNA from males will be somewhere between 0.5 and 1. Using this approach, the authors found that the Muller element A, the X in Drosophila, was an autosome ancestrally in diptera. Instead, what is now the autosomal dot chromosome in Drosophila (element F) was likely the X in ancestral species and remains a sex chromosome in several non-Drosophila families including *Tephritidae* (the other family commonly called fruit flies) and *Sarcophagidae* (the rather unpleasant family commonly known as flesh flies). These results demonstrate that sex chromosomes can revert back to autosomes, and that their differentiation does not have to be a dead end.

Now in this issue, Vicoso and Bachtrog have expanded their approach to characterize the turnover of sex chromosomes in 37 Dipteran species spanning over 250 million years of evolution [10]. They find that while Muller element F is indeed the ancestral X, multiple lineages substituted it with other chromosomes to evolve new X’s. The range of events observed is striking. Moreover, in some species the derived sex chromosomes are no longer heteromorphic, suggesting very recent acquisition of the sex-determining factor. In others, the sex chromosomes are slightly older, and likely in the process of differentiation. Overall, they find at least one example where all or part of each Muller element has become a sex chromosome. These findings and other recent studies raise many interesting issues, five of which we consider here.

### Loss of Dosage Compensation and Regained Diploidy

After the emergence of a new X, the old X has to reestablish diploidy in males and lose dosage compensation as it becomes an autosome. While there is little supporting evidence, meiotic nondisjunction (that is, the failure of two chromosomes to segregate during meiosis) is one mechanism that can restore diploidy in a single generation [9]. The simplest way for this to happen is for a sperm without the old X to fertilize an egg with two X’s that failed to segregate during meiosis in the mother.

As diploidy is restored, dosage compensation of the old X is not only unnecessary, but will result in deleterious imbalances of gene dosage. Therefore, dosage compensation of the old X needs to be eliminated. In the case of Drosophila, the dot chromosome is no longer dosage compensated, while the Muller A recruits the male-specific dosage compensation complex by using short motifs scattered throughout the chromosome. This change appears to be due, in part, to abandonment of ancestral machinery. In Drosophila, only the X and dot chromosomes have chromosome-specific targeting, the former by the dosage compensation machinery male-specific lethal (MSL) complex and the latter by the protein Painting of fourth (POF). Several lines of evidence have suggested that POF mediates dosage compensation of the dot ancestrally, but it appears to have no role in up-regulating the X in most species of Drosophila [11]. Curiously, the dot is also preferentially bound by SETDB1, a repressor that silences through histone methylation, potentially through interaction with POF [12]. This localization of SETDB1 may be a necessary function to prevent vestigial up-regulation of Muller F.

### Gain of Dosage Compensation

While it’s still unclear precisely how the Muller A acquired dosage compensation, several recent results give insight into how recruitment of dosage compensation proteins might rapidly evolve. On the neo-X chromosome of *Drosophila miranda*, a transposable element (TE) has provided the regulatory sequence to attract the dosage compensation complex [13]. TEs are small DNAs that can self-mobilize and rapidly increase their copy number by inserting throughout the genome. While typically considered selfish or parasitic elements, in this case a TE has provided a mutational source that serves an essential regulatory function. Another recent report discovered that small RNAs derived from a highly abundant noncoding repeat help assemble the dosage compensation complex in the fruit fly *D. melanogaster* [14]. The authors further suggest that other repeats may serve this function in closely related species. Interestingly, both the Muller element A and F (which is the ancestral sex chromosome) are heavily populated with dispersed repeats that show a high rate of turnover [15]. Perhaps such rapidly evolving repeats provide evolutionary flexibility to evolve dosage compensation.

### Sex Determination Signals

We outlined above the fluidity in sex determination signals, but the scale of karyotype change described by Viscoso and Bachtrog warrants renewed examination along the many evolutionary branches identified [10]. A first step would be to determine the chromosomal location of the Drosophila sex-determination regulatory genes, to test, for example, if any of the female determiners are on Muller F in species where it is the X. Likewise, other genes could potentially function as dominant male determiners on the Y chromosome. Zebrafish is another interesting animal for further study as it was recently reported that domesticated strains may have a different sex determination system compared to wild progenitors [16].

### Y Chromosome Evolution

Further analysis will be required to identify Y-linked genes, including potential male determiners. A comparative analysis of Drosophila Y chromosomes found a surprising amount of gene gain and suggested that the entire Y may turn over at a relatively high rate [17]. Recent studies on the mammalian Y found that although only a small set of the former autosomal genes are preserved, many of the remaining Y-linked genes display notable conservation, which is indicative of negative (purifying) selection [18,19]. Other studies found that primate Y’s also contain multicopy gene families that may have evolved under positive selection because they confer a selective advantage to males [20]. Moreover, a recent analysis of sequence variation among Drosophila species also suggests that both positive and negative selection can be detected among genes on the Drosophila Y [21]. Thus, the evolutionary forces acting on Y’s are clearly more complex than simple decay. Insight will also be gained from studies of other species, such as a recent survey of karyotypes among over 4,700 beetle species that documented extensive variation in presence and structure of Y chromosomes [22].

### Role of Heterochromatin

Highly differentiated Y chromosomes, such as in *Drosophila melanogaster*, are composed largely of noncoding, highly repetitive DNA that is kept in a silenced chromatin state known as heterochromatin. Assembling the protein-coding content of Y’s is challenging enough, and even less is known about the heterochromatic structure of Y’s even in intensively studied species such as humans. Yet there are hints that Y heterochromatin may have functional consequences. Studies have demonstrated that Y’s from geographically isolated populations can have different effects on gene expression and phenotypes such as immunity in *D. melanogaster* [23]. These results raise the possibility that variation in heterochromatin, such as copy number of transposable elements and simple-sequence repeats, is under selection. Recent advances in detecting and quantifying variation in simple-sequence repeats open new possibilities for investigating heterochromatin evolution [24,25]. We suggest that further studies of Y heterochromatin, particularly on young and neo-sex chromosomes, may reveal additional factors that bear upon our understanding of the surprising diversity and evolvability of sex chromosomes.

The study by Vicoso and Bachtrog paints a dynamic process in which the transition between autosomes and sex chromosomes is highly labile [10]. These transitions instigate alterations to key biological processes such as chromosome structure, sex determination, and gene regulation. Further studies will shed light on the mechanisms by which these processes evolve and, more generally, help us understand how organisms successfully navigate evolutionary transitions.

## References

[1] Larney C, Bailey TL, Koopman P (2014) Switching on sex: transcriptional regulation of the testis-determining gene Sry. Development 141: 2195–2205. 10.1242/dev.107052 24866114 PMC4034426

[2] Salz HK, Erickson JW (2010) Sex determination in Drosophila: The view from the top. Fly (Austin) 4: 60–70. 20160499 10.4161/fly.4.1.11277 PMC2855772

[3] Scott JG, Warren WC, Beukeboom LW, Bopp D, Clark AG, et al (2014) Genome of the house fly, Musca domestica L., a global vector of diseases with adaptations to a septic environment. Genome Biol 15: 466 25315136 10.1186/s13059-014-0466-3 PMC4195910

[4] Bachtrog D, Mank JE, Peichel CL, Kirkpatrick M, Otto SP, et al (2014) Sex determination: why so many ways of doing it? PLoS Biol 12: e1001899 10.1371/journal.pbio.1001899 24983465 PMC4077654

[5] Sánchez L (2008) Sex-determining mechanisms in insects. Int J Dev Biol 52: 837–856. 10.1387/ijdb.072396ls 18956315

[6] Verhulst EC, van de Zande L, Beukeboom LW (2010) Insect sex determination: it all evolves around transformer. Curr Opin Genet Dev 20: 376–383. 10.1016/j.gde.2010.05.001 20570131

[7] Matson CK, Zarkower D (2012) Sex and the singular DM domain: insights into sexual regulation, evolution and plasticity. Nat Rev Genet 13: 163–174. 10.1038/nrg3161 22310892 PMC3595575

[8] Ferrari F, Alekseyenko AA, Park PJ, Kuroda MI (2014) Transcriptional control of a whole chromosome: emerging models for dosage compensation. Nat Struct Mol Biol 21: 118–125. 10.1038/nsmb.2763 24500429 PMC4342042

[9] Vicoso B, Bachtrog D (2013) Reversal of an ancient sex chromosome to an autosome in Drosophila. Nature 499: 332–335. 10.1038/nature12235 23792562 PMC4120283

[10] Vicoso B, Bachtrog D (2015) Numerous transitions of sex chromosomes in Diptera. PLoS Biol 13: e1002078 10.1371/journal.pbio.1002055 25879221 PMC4400102

[11] Larsson J, Svensson MJ, Stenberg P, Mäkitalo M (2004) Painting of fourth in genus Drosophila suggests autosome-specific gene regulation. Proc Natl Acad Sci U S A 101: 9728–9733. 15210994 10.1073/pnas.0400978101 PMC470743

[12] Seum C, Reo E, Peng H, Rauscher FJ, Spierer P, Bontron S (2007) Drosophila SETDB1 is required for chromosome 4 silencing. PLoS Genet 3: e76 17500594 10.1371/journal.pgen.0030076 PMC1866353

[13] Ellison CE, Bachtrog D (2013) Dosage compensation via transposable element mediated rewiring of a regulatory network. Science 342: 846–850. 10.1126/science.1239552 24233721 PMC4086361

[14] Menon DU, Coarfa C, Xiao W, Gunaratne PH, Meller VH (2014) siRNAs from an X-linked satellite repeat promote X-chromosome recognition in Drosophila melanogaster. Proc Natl Acad Sci U S A. 10.1073/pnas.1410534111 PMC4246271 25368194

[15] Gallach M (2014) Recurrent turnover of chromosome-specific satellites in Drosophila. Genome Biol Evol 6: 1279–1286. 10.1093/gbe/evu104 24846631 PMC4079201

[16] Wilson CA, High SK, McCluskey BM, Amores A, Yan YL, et al (2014) Wild sex in zebrafish: loss of the natural sex determinant in domesticated strains. Genetics 198: 1291–1308. 10.1534/genetics.114.169284 25233988 PMC4224167

[17] Koerich LB, Wang X, Clark AG, Carvalho AB (2008) Low conservation of gene content in the Drosophila Y chromosome. Nature 456: 949–951. 10.1038/nature07463 19011613 PMC2713029

[18] Bellott DW, Hughes JF, Skaletsky H, Brown LG, Pyntikova T, et al (2014) Mammalian Y chromosomes retain widely expressed dosage-sensitive regulators. Nature 508: 494–499. 10.1038/nature13206 24759411 PMC4139287

[19] Cortez D, Marin R, Toledo-Flores D, Froidevaux L, Liechti A, et al (2014) Origins and functional evolution of Y chromosomes across mammals. Nature 508: 488–493. 10.1038/nature13151 24759410

[20] Hughes JF, Skaletsky H, Pyntikova T, Graves TA, van Daalen SKM, et al (2010) Chimpanzee and human Y chromosomes are remarkably divergent in structure and gene content. Nature 463: 536–539. 10.1038/nature08700 20072128 PMC3653425

[21] Singh ND, Koerich LB, Carvalho AB, Clark AG (2014) Positive and purifying selection on the Drosophila y chromosome. Mol Biol Evol 31: 2612–2623. 10.1093/molbev/msu203 24974375 PMC4166921

[22] Blackmon H, Demuth JP (2014) Estimating tempo and mode of Y chromosome turnover: explaining Y chromosome loss with the fragile Y hypothesis. Genetics 197: 561–572. 10.1534/genetics.114.164269 24939995 PMC4063915

[23] Lemos B, Branco AT, Hartl DL (2010) Epigenetic effects of polymorphic Y chromosomes modulate chromatin components, immune response, and sexual conflict. Proc Natl Acad Sci U S A 107: 15826–15831. 10.1073/pnas.1010383107 20798037 PMC2936610

[24] Aldrich JC, Maggert KA (2014) Simple quantitative PCR approach to reveal naturally occurring and mutation-induced repetitive sequence variation on the Drosophila Y chromosome. PLoS ONE 9: e109906 10.1371/journal.pone.0109906 25285439 PMC4186871

[25] Wei KH-C, Grenier JK, Barbash DA, Clark AG (2014) Correlated variation and population differentiation in satellite DNA abundance among lines of *Drosophila melanogaster* . Proc Natl Acad Sci U S A 111: 18793–18798. 10.1073/pnas.1421951112 25512552 PMC4284603

